# Long term high glucose exposure induces premature senescence in retinal endothelial cells

**DOI:** 10.3389/fphys.2022.929118

**Published:** 2022-08-26

**Authors:** Pietro Maria Bertelli, Edoardo Pedrini, David Hughes, Shannon McDonnell, Varun Pathak, Elisa Peixoto, Jasenka Guduric-Fuchs, Alan W Stitt, Reinhold J. Medina

**Affiliations:** Wellcome-Wolfson Institute for Experimental Medicine, School of Medicine, Dentistry, and Biomedical Sciences, Faculty of Medicine, Health, and Life Sciences, Queen’s University Belfast, Belfast, United Kingdom

**Keywords:** diabetic retina, senescence, retinal microvasculature, diabetic retina complications, vascular biology, senescence-associated secretory phenotype, endothelial cell

## Abstract

**Purpose:** Features of cellular senescence have been described in diabetic retinal vasculature. The aim of this study was to investigate how the high glucose microenvironment impacts on the senescence program of retinal endothelial cells.

**Methods:** Human retinal microvascular endothelial cells were cultured under control and high glucose conditions of 5 mM and 25 mM D-glucose, respectively. Isomeric l-glucose was used as the osmotic control. Cells were counted using CASY technology until they reached their Hayflick limit. Senescence-associated β-Galactosidase was used to identify senescent cells. Endothelial cell functionality was evaluated by the clonogenic, 3D tube formation, and barrier formation assays. Cell metabolism was characterized using the Seahorse Bioanalyzer. Gene expression analysis was performed by bulk RNA sequencing. Retinal tissues from db/db and db/+ mice were evaluated for the presence of senescent cells. Publicly available scRNA-sequencing data for retinas from Akimba and control mice was used for gene set enrichment analysis.

**Results:** Long term exposure to 25 mM D-Glucose accelerated the establishment of cellular senescence in human retinal endothelial cells when compared to 5 mM D-glucose and osmotic controls. This was shown from 4 weeks, by a significant slower growth, higher percentages of cells positive for senescence-associated β-galactosidase, an increase in cell size, and lower expression of pRb and HMGB2. These senescence features were associated with decreased clonogenic capacity, diminished tubulogenicity, and impaired barrier function. Long term high glucose-cultured cells exhibited diminished glycolysis, with lower protein expression of GLUT1, GLUT3, and PFKFB3. Transcriptomic analysis, after 4 weeks of culture, identified downregulation of *ALDOC, PFKL,* and *TPI1,* in cells cultured with 25 mM D-glucose when compared to controls. The retina from db/db mice showed a significant increase in acellular capillaries associated with a significant decrease in vascular density in the intermediate and deep retinal plexuses, when compared to db/+ mice. Senescent endothelial cells within the db/db retinal vasculature were identified by senescence-associated β-galactosidase staining. Analysis of single cell transcriptomics data for the Akimba mouse retina highlighted an enrichment of senescence and senescence-associated secretory phenotype gene signatures when compared to control mice.

**Conclusion:** A diabetic-like microenvironment of 25 mM D-glucose was sufficient to accelerate the establishment of cellular senescence in human retinal microvascular endothelial cells.

## 1 Introduction

Diabetic retinopathy is a frequent microvascular complication that leads to vision impairment and blindness. The vision-threatening complications associated with diabetic retinopathy are diabetic macular edema (DME) and proliferative diabetic retinopathy (PDR), whose pathophysiology are driven by blood retinal barrier breakdown, or pathological neovascularization, respectively (Duh et al., 2017). While all components of the neurovascular unit are affected by diabetes (Gardner and Davila, 2017), damage and loss of the retinal microvasculature plays a major role in the development of diabetic retinopathy and furthermore, the current clinical classification for diabetic retinopathy is based on retinal vascular lesions (Stitt et al., 2016). This retinal microvascular dysfunction has been associated with hyperglycemia-induced metabolic abnormalities leading to oxidative stress (Kang and Yang, 2020). In addition, it has been recently shown that pathological vessels in diabetic retinopathy exhibit some features of endothelial senescence (Crespo-Garcia et al., 2021).

Cellular senescence is an irreversible state of cell cycle arrest, characterized by multiple phenotypic changes and impaired cell functionality (Hernandez-Segura et al., 2018). Cellular senescence can be induced by several stimuli, which determine senescence subtypes such as, replicative senescence, stress-induced senescence, and oncogene-induced senescence (Munoz-Espin and Serrano, 2014). The main hallmarks of senescence include both morphological and molecular alterations (Gorgoulis et al., 2019). Senescent cells show bigger and irregular shape, organelle enlargement and positivity for senescence-associated beta-galactosidase (SA-ß-Gal). They are also characterized by DNA damage, metabolic dysfunction, and the senescence-associated secretory phenotype (SASP), including inflammatory, growth and tissue remodeling factors (Lopez-Otin et al., 2013; Hernandez-Segura et al., 2018).

Cellular senescence has been associated with the pathogenesis of several age-related diseases, including diabetes (Tian and Li, 2014; Palmer et al., 2015). Interestingly, the diabetic milieu and the SASP were suggested as drivers of retinal senescence both *in vitro* and *in vivo* (Oubaha et al., 2016; Thounaojam et al., 2019; Liu et al., 2020). While these studies have established the presence of senescent vasculature in diabetic retinas, further studies are needed to dissect the molecular biology of the diabetic microenvironment on retinal endothelial cells. Here, we investigated the senescence program of human retinal microvascular endothelial cells cultured under high glucose conditions, akin to diabetes, and found that a constant exposure to 25 mM D-glucose is sufficient to trigger premature endothelial senescence. We show that this premature endothelial senescence phenotype induced by diabetic conditions exhibited impaired glycolysis. In addition, we identified SA-ß-Gal positive senescent endothelial cells within the diabetic retinal vasculature.

## 2 Materials and methods

### 2.1 Cell culture

Human Retinal Microvascular Endothelial cells (HRMECs) were purchased from Innoprot. Cells were derived from adult eyes of male donors. HRMECs were cultured on flasks coated with fibronectin from human plasma (F2006, Sigma-Aldrich) and in Endothelial Cell Medium (ECM, Innoprot) composed of 5% Fetal Bovine Serum (FBS), Endothelial Cell Growth Supplements (ECGS) and penicillin/streptomycin. HRMECs were grown in normal (5 mM D-glucose) and high-glucose (25 mM D-Glucose) conditions. Cells were cultured in 5 mM D-glucose plus 20 mM l-Glucose as the osmotic control. HRMECs between passage 4 (P4) and P5 were used as early passage (ep) controls. Cells were treated with high-glucose conditions for 4 weeks. RNA and protein were sampled after 4-week-culture under high glucose conditions when HRMECs were P11-P16 and defined as late passage (lp). Hayflick limit was reached at ≥ P20. To define HRMEC proliferation capacity *in vitro*, growth curves were generated. Cells were passaged every 3–5 days and seeded at a known density (2 × 10^4^/ml) to measure population doubling level (PDL) at each passage, until they reached Hayflick limit. The CASY cell counter analyzer (Cambridge Bioscience) was used for cell counts at every passage.

### 2.2 Senescence-associated β-galactosidase (SA-β-gal) staining

β-galactosidase activity at pH 6 was investigated using the Senescence β-Galactosidase Staining Kit (Cell Signaling Technology). Cells or mouse retinal tissues were fixed for 15 min with 1X fixative solution and stained with staining solution overnight in a 37°C incubator (no CO_2_). Cells and retinal tissues were imaged using the Leica DMi8 microscope.

### 2.3 Immunocytochemistry

HRMECs were grown on fibronectin-coated glass coverslips and fixed using 100% ice-cold methanol for 20 min at -20°C. Blocking was performed with 5% goat serum (G9023, Sigma-Aldrich) in PBS containing 0.1% Triton X-100 (vol/vol, PBST) for 1 h at room temperature. Cells were stained with primary antibody for VE-Cadherin (ab33168, Abcam, 1:200) overnight at 4°C. After washing with PBST, goat anti-rabbit AF568 (A11036, Life Technologies, 1:500) secondary antibody was applied for 1 h at room temperature. Coverslips were mounted on glass slides using Vectashield with DAPI (Vector Laboratories, H-1200). Cells were imaged using the Leica DMi8 microscope. Fiji-ImageJ2 software (Schindelin et al., 2012) and R version 4.0.4 (R Core Team, 2021) were used for analysis of cell size.

### 2.4 Protein extraction and western blotting

Cells were lyzed in 1X RIPA buffer supplemented with EDTA, protease and phosphatase inhibitors (Thermo Fisher). Protein concentration was measured using the Pierce BCA Protein Assay Kit (Thermo Fisher) as per manufacturer protocol, 20 µg of protein was loaded onto SDS polyacrylamide gels. After electrophoresis, proteins were transferred to a PVDF membrane. Membranes were blocked for 1 h in 5% non-fat dry milk (Santa Cruz Biotechnology) in TBS with 0.1% Tween-20 (vol/vol, TBST), followed by incubation with primary antibodies overnight at 4°C. Primary antibodies were made in 1X Clear Milk (ThermoFisher Scientific). After washing with TBST, horseradish peroxidase conjugated (HRP) secondary antibodies (Biorad), made in 1X Clear Milk, were applied at 1:3000 dilution for 1 h at room temperature. After washing in TBST, blots were developed using chemiluminescence HRP substrate (Biorad) and imaged with G:BOX instrument (Syngene). The immunoblots were quantified and analyzed using ImageJ software (Schindelin et al., 2012). Antibodies for p-Rb (9308), SIRT-1 (8469), HMGB2 (14163), HK-2 (2867), LDHA (3582), PFKFB3 (13123) and β-actin (5125) were used at 1:1000 concentration and supplied from Cell Signaling Technologies. Antibodies for GLUT1 (NB110-39113), GLUT3 (NBP1-89762) and PAI-1 (NBP1-19773) were purchased from Novus Biologicals and used at 1:500 and 1:1000 dilution, respectively.

### 2.5 RNA extraction

Total RNA was isolated using the Maxwell RSC simplyRNA Cells Kit (Promega) following manufacturer protocol. Concentration of RNA was measured by Nanodrop ND-100 Spectrophotometer (Nanodrop Technologies) and Qubit 2.0 Fluorometer (Life Technologies). Purity was determined by Nanodrop ND-100 Spectrophotometer, where absorbance ratios 260/230 and 260/280 were close to 2. The Bioanalyzer 2100 (Agilent) was utilized to determine RNA integrity.

### 2.6 Barrier permeability assay

The experimental system utilized was the xCELLigence Real-Time Cell Analyzer (RTCA). E-Plate 96 (RTCA, Agilent) was coated with fibronectin from human plasma (F2006, Sigma-Aldrich). After baseline measurement, 20,000 cells in 200 µl of ECM were pipetted in each well. Cells were let to settle at room temperature in the hood for 30 min before placing plate into the machine. Electric resistance was measured every 15 min for 15–18 h, until barrier was fully formed. Barrier permeability was disrupted by treating HRMECs monolayer with 50 ng/ml VEGF (293-VE-010/CF, R&D Systems). Electrical resistance was measured every minute for 2 h and every 5 min for 6–8 h. Decrease in barrier capacity was assessed to analyze HRMECs barrier permeability. RTCA Data Analysis Software 1.0 was used for analysis.

### 2.7 Clonogenic assay

HRMECs were seeded in 6-well plates in 2 ml of media containing 200 cells per well. Plates were coated with fibronectin from human plasma (F2006, Sigma-Aldrich). HRMECs were left for 7 days, with media changed every 2 days. Experimental end point was reached when colonies formed were visible but remained separate from each other. To analyze colony formation, crystal violet (Cat #C0775, Sigma) was added to each well for 1 h room temperature. After 1 h the crystal violet solution was removed and each well was washed with PBS to remove excess stain, before the plates were submerged in water and then left to dry overnight. The entire well was imaged using the EVOS Cell Imaging system (Thermo Fisher). Analysis of the colonies formed was performed using Fiji-ImageJ2 software (Schindelin et al., 2012).

### 2.8 3D tube formation assay

The angiogenesis μ-Slide (Ibidi) was used to assess 3D tube formation capacity. An HRMEC suspension was mixed in a 2:3 ratio with Matrigel (Corning) and dispensed into slide to form a 10 μl Matrigel droplet containing 20,000 cells (final density of 2 million HRMECs/ml). Angiogenesis μ-Slides were left in the incubator for 30 min to enable Matrigel polymerization, and then covered with 50 μl of ECM media (Innoprot). ECM was supplemented to a final concentration of 25 mM D-glucose for high glucose conditions. ECM supplemented with 20 mM l-Glucose was used as the osmotic control. Tube-like structures were assessed after 24 h, using the EVOS Cell Imaging system (Thermo Fisher). Analysis of the tube area was performed using Fiji‐ImageJ2 software (Schindelin et al., 2012).

### 2.9 Glycolysis assays

Metabolic assessment was carried out using the Seahorse XFe96 analyzer (Agilent). 96 well-plate was coated with fibronectin from human plasma (F2006, Sigma-Aldrich) and HRMECs were seeded at a density of 20,000 in 80 µl per well overnight. Sensor cartridge was hydrated overnight as per manufacturer protocol. 20 ml of Calibrant were also equilibrated at 37°C with no CO_2_. The following day, Assay media (103575–100, Agilent), cells and injections were prepared for experiment as per manufacturer protocol. Glycolysis Stress kit (103020–100, Agilent) and Energy Phenotype kit (103325–100, Agilent) were used for metabolic assessment in HRMECs. Seahorse Wave Controller Software 2.4 was used for analysis and report generator.

### 2.10 2-NBDG glucose uptake

Glucose uptake was measured using 2-NBDG (Sigma-Aldrich) at a final concentration of 200 µM. HRMECs were pelleted and stained for 15 min at 37°C in 100 µl 2-NBDG solution prepared in Flow cytometry staining buffer (FACS, eBioscience). After staining, cells were washed with 1 ml of FACs buffer and centrifuged at 300 RCF for 8 min at room temperature. Pellets were resuspended in 500 µl FACs buffer before running samples using an Attune NxT flow cytometer (ThermoFisher Scientific). Unstained samples were used as controls. 100,000 events were recorded for each sample. Data were analyzed using FlowJo (Oregon, United States).

### 2.11 Glucose consumption measurement

HRMECs were seeded at the density of 10,000 cells per well in a 24-well plate coated with fibronectin from human plasma (Sigma Aldrich). Glucose levels were measured in the ECM media with a GlucCell Glucose Monitoring System (CESCO Bioproducts) before cell seeding. HRMECs were let to grow for 72 h, and glucose level was measured again to calculate glucose consumption. One well without cells was used as background control, to account for technical changes in relation to temperature and pH. Results were then normalized to total cell number, to provide data on glucose consumption per cell.

### 2.12 Glycogen content assay

HRMECs were pelleted and counted. Cell pellet was washed in cold 1X PBS and resuspended in double-distilled water (ddH2O), as per protocol (ab65620, Abcam). Suspensions were then homogenized by pipetting up and down a couple of times and boiled at 95°C for 10 min to inactivate enzymes. After centrifugation for 10 min at 4°C at 18,000 g, supernatant was collected, and colorimetric assay was then performed following manufacturer protocol. Output was measured using the Varioskan LUX plate reader (ThermoFisher Scientific) at optical density (OD) 570 nm. Results were then normalized to total cell number, to provide data on glycogen content per cell.

### 2.13 Hexokinase activity assay

HRMECs were pelleted and counted. Cell pellet was washed in cold 1X PBS and resuspended in Assay buffer, as per protocol (ab136957, Abcam). Suspensions were then homogenized by pipetting up and down a couple of times. After centrifugation for 5 min at 4°C at 12,000 rpm, supernatant was collected, and colorimetric assay was then performed following manufacturer protocol. Output was measured using the Varioskan LUX plate reader (ThermoFisher Scientific) at optical density (OD) 450 nm. Output was measured at 30 and 60 min. Values obtained at 60 min were subtracted from the values obtained at 30 min. Results were then normalized to total cell number, to provide data on hexokinase activity per cell.

### 2.14 RNA sequencing

Total RNA was extracted from 4-weeks high glucose-treated HRMECs and passage matched control HRMECs. Extraction was performed using the Maxwell RSC simplyRNA Cells Kit (Promega) and a Maxwell RSC instrument (Promega) following the manufacturer’s instructions. Three biological replicates were sent for each condition. Libraries were prepared using TruSeq stranded mRNA-seq library. Samples were sequenced on an Illumina NovaSeq instrument (Illumina) to yield at least 50M pair reads per sample 50 PE. Data were normalized and differential expression of genes identified using DESeq. GSEA was performed using the fgsea package in R as an algorithm for pre-ranked GSEA using cumulative statistic calculation. Data deposited to the NCBI Gene Expression Omnibus (GEO) database under GEO: GSE199548. Raw single cell RNAseq data for the Akimba and control mouse model were downloaded from ArrayExpress (E-MTAB-9061) and re-analyzed following the workflow presented in the paper.

### 2.15 Animals and vasculature staining

All animal experiments are conformed to Home Office regulations and were approved by the Animal Welfare Ethical Review Body (AWERB) of Queen’s University Belfast, in accordance with the United Kingdom Animals (Scientific Procedures) Act 1986. A total of 14 inbred, age matched BKS.Cg-Dock7m+/+Leprdb/J mice were divided into two groups of 7 non-diabetic controls (db/+) and 7 (db/db) diabetic mice. Mice were fed normal rodent chow and water was available *ad libitum*. Once the duration of diabetes was 6–9 months, age matched mice were euthanized, and eyes were immediately enucleated. Freshly dissected eyes were fixed in 4% paraformaldehyde (pH 7.2) for 2 h at room temperature and were stored in PBS at 4°C. For retinal vasculature analysis, the cornea and lens were removed, and the optic cup was whole mounted by separating into 4 quadrants. After dissection, the retinas were placed into wells of a 96 well-plate and blocked overnight at 4°C using permeabilization/block (Perm/Block) buffer [PBS supplemented with 0.5% Triton X-100, 1% Goat serum (G9023, Sigma-Aldrich) and 0.1 mM CaCl_2_]. Retinas were then stained with 20 µg/ml Isolectin B4 (L21240, Sigma-Aldrich) in Perm/Block buffer at 4°C for 5 days on a rocker. After a wash in Perm/Block buffer overnight at 4°C, AF488 Streptavidin-conjugated secondary antibody (S32354, Life Technologies, 1:500) was applied overnight at 4°C in the dark. Retinas were then washed multiple times in Perm/Block buffer every 30 min for 4 h in dark and mounted on glass slides using Vectashield (Vector Laboratories, H-1000). The Leica SP5 confocal microscope was used for image acquisition and images were analyzed using the Fiji-ImageJ2 and AngioTool plug-in (Zudaire et al., 2011; Schindelin et al., 2012).

### 2.16 Retinal tissue staining for acellular capillaries

Assessment of acellular capillaries was performed by Collagen IV staining in flat mounted retinas already stained with isolectin as described in Section 2.14. Mouse retinas were blocked in a 96-well plate containing Perm/Block overnight at 4°C on a rocker. The Perm/Block buffer was then replaced with a fresh Perm/Block buffer containing primary rabbit monoclonal collagen IV antibody (BP5031, Origene, 1:200) and incubated at 4°C on a gentle rocker for 5 days. The retinas were washed three times in Perm/Block buffer and incubated with secondary goat anti-rabbit AF568 antibody (A11036, Life Technologies, 1:500) overnight at 4°C. We then performed three washes with fresh Perm/Block buffer and mounted the retinas on slides using Vectashield antifade mounting medium (Vector Laboratories H-1000). Images were acquired using the Leica Stellaris-5 confocal microscope (Leica Microsystems), and quantification of acellular capillaries was performed using Fiji-ImageJ2.

### 2.17 Statistical analysis

GraphPad Prism was used for data analysis. Data are represented as mean ± SEM. Two-tailed paired or unpaired t-test was used to test significance when comparing two groups, depending on experimental design. One-way ANOVA, with Tukey’s post-hoc analysis, were used when more than two groups were compared. Statistically significant values were set at *p*-value < 0.05.

## 3 Results

### 3.1 Accelerated cellular senescence in human retinal microvascular endothelial cells cultured under high glucose conditions

**FIGURE 1:**
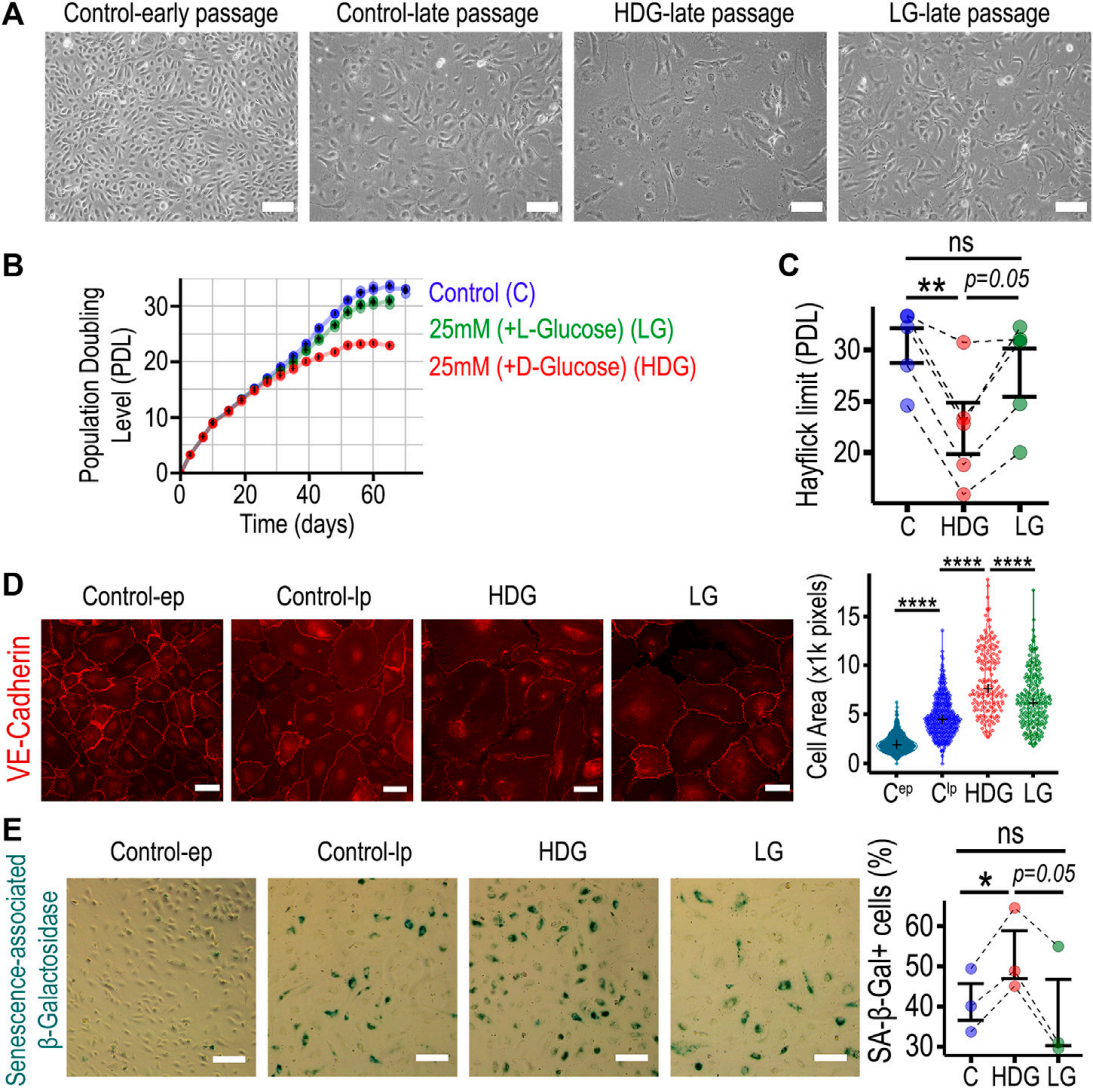
HRMECs undergo premature cellular senescence when cultured long term with 25 mM D-Glucose. **(A)** Phase-contrast 10X microscopy images of HRMECs at early passage (P4-P5), late passage (P11-P16), and experimental groups of late passage HRMECs treated with high glucose conditions (HDG or LG). Scale bar: 150 µm. **(B)** Growth curves for three experimental groups: control group cultured under 5 mM D-glucose, osmotic control topped up to 25 mM with l-glucose, and high glucose with 25 mM D-glucose. HRMECs were counted after every passage with automated CASY cell counter, cumulative population doublings calculated and plotted against time in culture. **(C)** For maximal cell number expansion, population doubling level reached at the Hayflick limit was used for statistical comparison. **(D)** Representative 20X images of VE-cadherin immunostaining in HRMECs after 4 weeks in culture to evaluate cell surface area using ImageJ. Scale bar: 50 µm **(E)** Representative 10X phase-contrast images of senescence-associated β-Galactosidase staining in HRMECs after 4 weeks in culture to identify and quantify senescent endothelial cells. Scale bar: 150 µm. *p < 0.05, **p < 0.01, ****p < 0.0001, ns: not significant. One-way ANOVA, with Tukey’s post-hoc analysis was used. PDL, Population doubling level; C, control (5 mM D-Glucose); LG, 25 mM (+L-glucose); HDG, 25 mM (+D-glucose); ep, early passage; lp, late passage.

To mimic the hyperglycemic diabetic microenvironment *in vitro*, human retinal microvascular endothelial cells (HRMECs) were cultured in 25 mM D-Glucose as high D-Glucose (HDG). As controls, we used 5 mM D-glucose (C). We also added 20 mM l-glucose to C as an osmotic control (LG). HRMECs were counted at every passage using a CASY counter and growth curves generated. In addition, the CASY technology was used to evaluate cell size and viability. HRMECs cultured under HDG did not exhibit changes in cell viability; however, they showed slow growth evident in phase-contrast images when compared to controls (Figure 1A). This was associated with a significant increase in cell diameter when compared to age-matched controls (Supplementary Figure S1). Importantly, HRMECs cultured under HDG exhibited a significant decrease in the population doubling level (PDL) from 4 weeks onwards (Figure 1B and Supplementary Figure S2). The Hayflick limit in HRMECs in the HDG group was significantly lower than control C group (Figure 1C). Cell passage was associated with an increase in cell area, and the 4-weeks HDG treatment led to a further significant increase when compared to similar aged cells cultured under control conditions (Figure 1D). We used SA-β-galactosidase staining as a senescence marker and confirmed, as expected, that there was negligible number of senescent cells in the control early passage group (Figure 1E). Interestingly, we found that there were significantly higher numbers of SA-β-Gal positive cells in the HDG group when compared to controls C-lp and LG, while there was no difference between the osmotic control LG and C-lp (Figure 1E). Furthermore, protein analysis to evaluate expression of senescence-related proteins in HRMECs cultured for 4-weeks under HDG, when compared to C-lp, showed a decrease in pRb, HMGB2, and Sirt1; an increase in p53; however, there was no difference in p21, p16, and PAI-1 expression (Supplementary Figure S3). Taken together, these results suggest that long term (over 4 weeks) 25 mM D-Glucose promoted an accelerated cellular senescence program in HRMECs, characterized by slower growth, larger cell size, higher positivity for SA-β-galactosidase staining, and lower Hayflick limit. Expression changes for senescence-related proteins were consistent for pRb and HMGB2, but not for p21 and p16.

### 3.2 Endothelial function is impaired in HRMECs cultured with 25 mM D-glucose for 4 weeks

**FIGURE 2:**
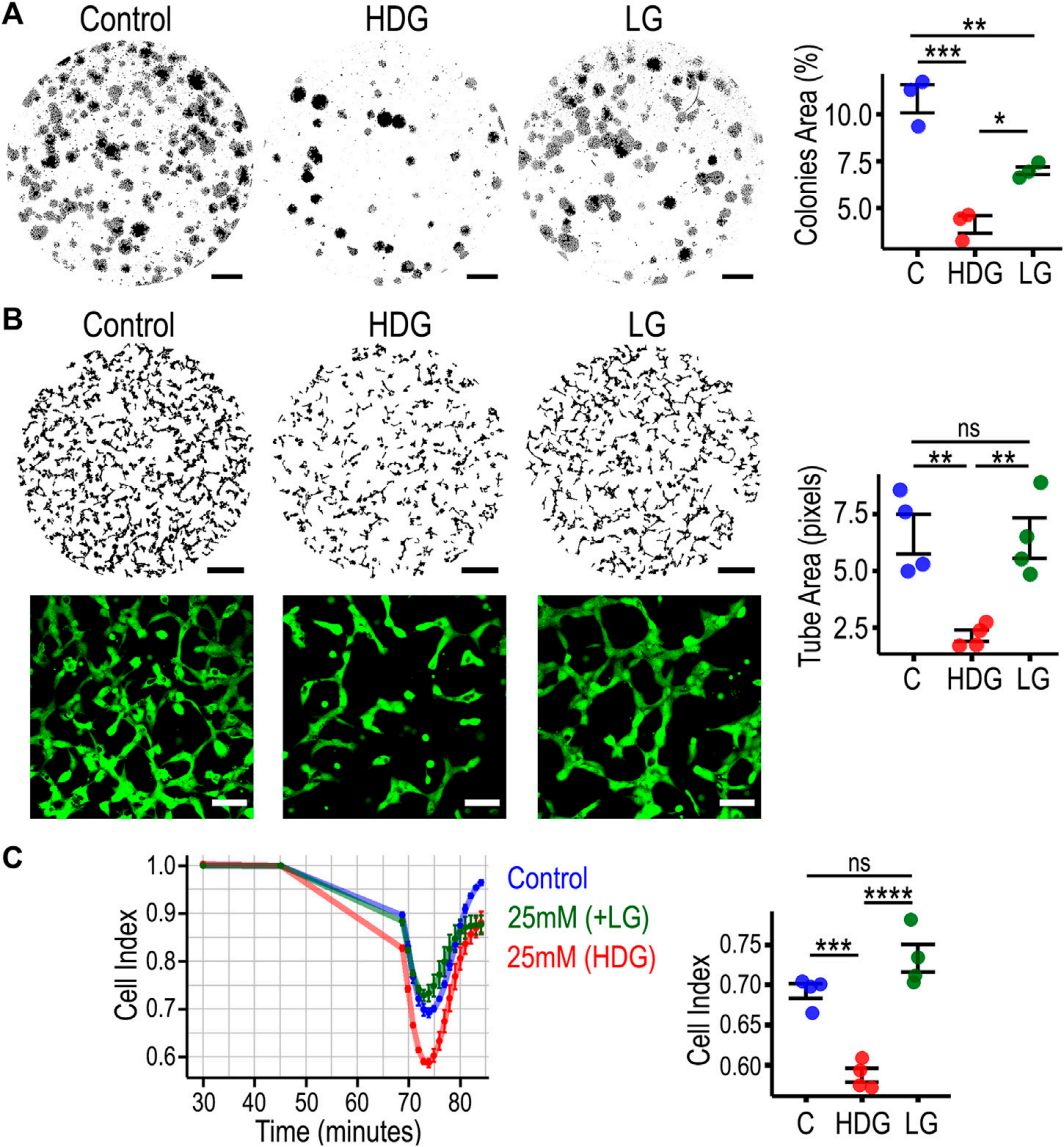
High glucose-induced premature senescent HRMECs display impairment in endothelial cell function. **(A)** Representative images of clonogenic assay and quantification with statistical comparison. 10X images were stitched together and the whole well of a 6-well plate is shown. Scale bar: 4 mm. **(B)** Representative images of 3D tube formation assay and quantification with statistical comparison. 10X images were stitched together and the whole well of a 96-well plate is shown. Scale bar: 500 µm. For 20X images, cells were stained with calcein in green. Scale bar: 100 µm. **(C)** Cell index traces from xCELLigence system to evaluate barrier function. VEGF was added at 45 min as the stimuli to disrupt endothelial barrier. Statistical comparison performed at 74 min as the point with highest decrease in electrical resistance. *p < 0.05, **p < 0.01, ***p < 0.001, ****p < 0.0001, ns: not significant. One-way ANOVA, with Tukey’s post-hoc analysis was used. C, control; LG, 25 mM (+L-glucose); HDG, 25 mM (+D-glucose).

As cellular senescence is associated with impaired cell functionality, we performed *in vitro* assays to evaluate endothelial clonogenicity, 3D tube-forming capacity, and barrier properties. Clonogenic capacity was significantly diminished in HRMECs exposed to HDG for 4 weeks, when compared to passage-matched control cells (Figure 2A). The osmotic control LG also decreased clonogenicity but with a lesser effect size. In the 3D angiogenesis assay, HRMECs cultured with HDG showed significant impairment in 3D tube formation capacity when compared to other experimental groups (Figure 2B), and there was no difference between C and osmotic control LG. Furthermore, we did not observe morphological changes when endothelial tube structures from HDG were compared to LG using higher magnification (Figure 2B). Endothelial barrier permeability was measured as Cell Index using the xCELLigence system. After adding VEGF as an inducer of endothelial barrier disruption, we noted that HRMECs cultured under HDG exhibited the highest drop in cell index when compared to both controls (Figure 2C). These data demonstrated that HRMECs cultured under 25 mM D-Glucose for 4 weeks already exhibited significant diminished endothelial function when compared to controls.

### 3.3 Cell metabolic profiling identified diminished glycolytic capacity in HRMECs cultured under high glucose conditions

**FIGURE 3:**
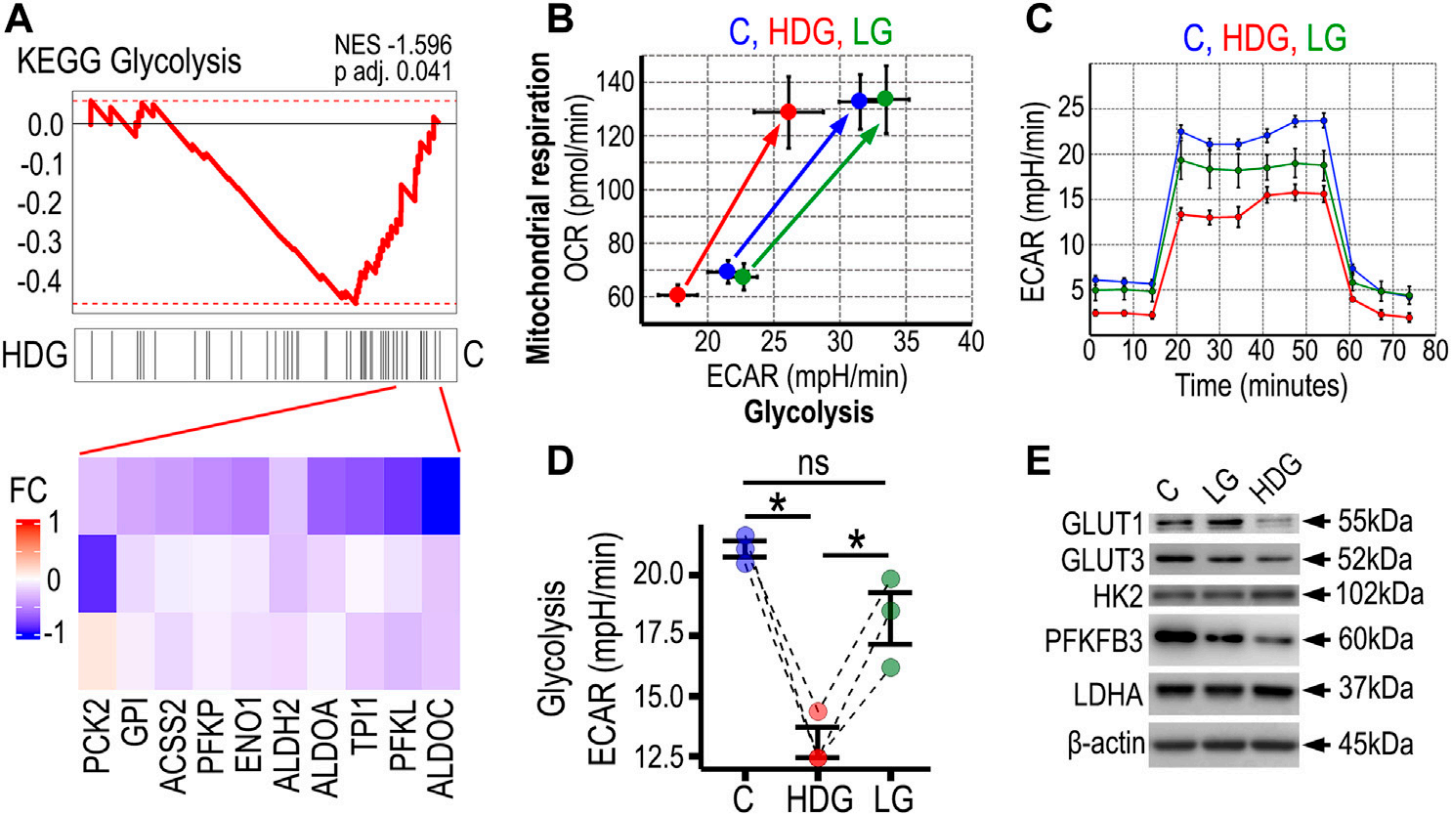
High glucose-induced premature senescent HRMECs exhibit diminished glycolysis. **(A)** Gene Set Enrichment Analysis for Glycolysis gene signature. Transcriptome data from bulk RNA sequencing of three biological replicates of HRMECs cultured with 25 mM D-glucose vs. the 5 mM control at 4 weeks of culture. Gene signature from KEGG Glycolysis was used for the gene set enrichment analysis (GSEA). Heatmaps below depict the leading edges for transcripts responsible for major differences. NES: Normalized Enrichment Score. p adj: adjusted *p* value. **(B)** Energy phenotype plot from Seahorse XFe96 analyzer depicting assessment of glycolysis as extracellular acidification rate (ECAR) and mitochondrial respiration as oxygen consumption rate (OCR) at baseline and stressed phenotypes. **(C)** Glycolysis stress assay to characterize glycolysis in HRMECs under different culture conditions. Injections in the assay were glucose at 20 min, oligomycin at 40 min, and 2-DG at 60 min. **(D)** Statistical comparison of basal glycolysis measures using Seahorse Glycolysis stress assay in three biological replicates. **(E)** Western blot analysis to compare expression of glycolysis-related proteins. β-actin was used as the loading control. *p < 0.05, ns: not significant. One-way ANOVA, with Tukey’s post-hoc analysis was used. C, control in blue; LG in green, 25 mM (+L-glucose); HDG in red, 25 mM (+D-glucose).

To gain insight into the molecular mechanisms driving the impairment of endothelial cell function after 4 weeks exposure to HDG, we extracted total RNA from all the experimental groups and performed RNA sequencing. We found that the cell passage was a major driver of senescence, and Etoposide-induced senescent HRMECs (positive control for cell senescence) showed the largest changes at the transcriptome level as shown in PCA plot (Supplementary Figure S4). When performing a targeted gene set enrichment analysis for glycolysis comparing HRMECs from HDG and C groups, we found a negative enrichment (NES: 1.596, *p*-value: 0.041) for a KEGG glycolysis and gluconeogenesis signature (M11521) in high glucose HRMECs (Figure 3A). These transcriptomics results suggested impaired glycolysis in HRMECs exposed to HDG for 4 weeks. To further investigate how the high glucose condition was affecting cellular metabolism in HRMECs that were undergoing premature senescence and impaired functionality, we performed Seahorse Real-Time cell metabolic analysis after 4 weeks in HDG culture. Both glycolysis and mitochondrial respiration, under baseline and stress conditions, were evaluated using the Energy Phenotype kit and measured as extracellular acidification rate (ECAR) and oxygen consumption rate (OCR), respectively. We found that both under basal and stress readouts, glycolysis was significantly decreased in HDG group when compared to C and LG (Figure 3B). When mitochondrial respiration was assessed, we found no differences among the groups. To gain further understanding surrounding these significant changes in glycolysis, we used the Seahorse Glycolysis Stress Kit (Figure 3C) and confirmed that HRMECs in the HDG group showed significantly lower glycolysis activity than C and LG groups (Figure 3D). Additionally, we investigated protein expression and found that HRMECs cultured under HDG for 4 weeks, exhibited lower expression of the glucose transporters, GLUT1 and GLUT3, and of the glycolytic enzyme PFKFB3, when compared to controls. No changes were seen in HK-2 and LDHA expression (Figure 3E). As oxidative stress plays a central role in the pathogenesis of diabetic retinopathy and is considered a process that links with both endothelial dysfunction and glycolysis, we evaluated SODs expression in HRMECs in our model. Interestingly, we found a decrease in SOD1 but not SOD2 in HRMECs exposed to HDG (Supplementary Figure S5). In addition, we performed biochemical assays including glucose consumption, glucose uptake, hexokinase activity, and glycogen content. Results were variable among the biological replicates, and although we were limited by the low statistical power of the study, we found an increase in glucose consumption and uptake in two out of three HRMECs in the HDG group. There was no reproducible result in relation to hexokinase activity; however, we found that there was an increase in intracellular glycogen content in four out of the five biological replicates for HRMECs cultured under HDG when compared to controls (Supplementary Figure S6A-E). These data underscored the glycolytic impairment in HRMECs that are exposed to a diabetic-like environment of 25 mM D-glucose, however it also highlighted that HRMECs from different donors had different responses across the biochemical assays.

### 3.4 Diabetic mice retinas exhibit accumulation of senescent endothelial cells

**FIGURE 4:**
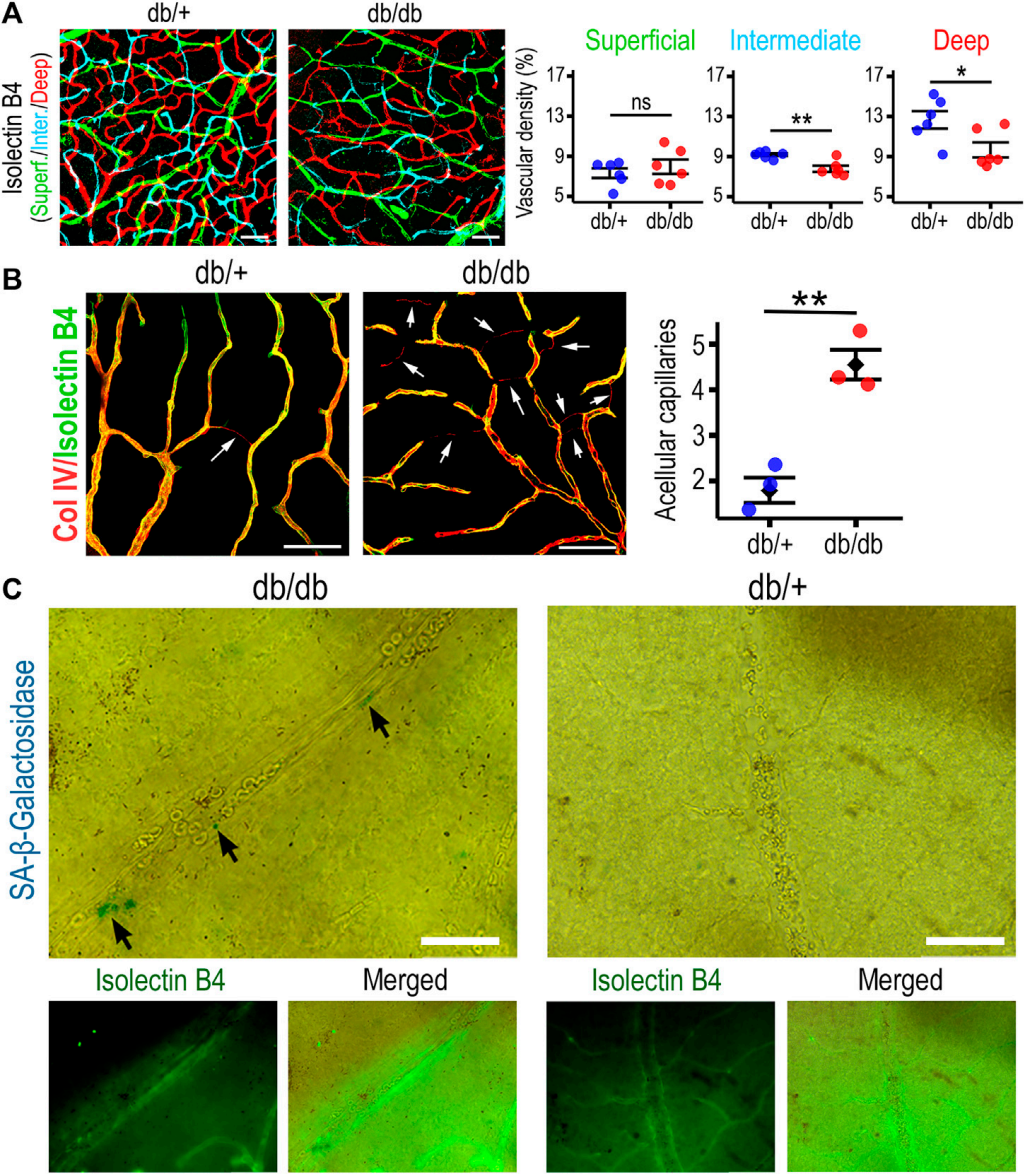
Db/db retinas exhibit vascular attrition and accumulation of senescent cells in vasculature. **(A)** Retinal tissue from 6–9-months mice stained with Isolectin B4 to identify vasculature, and pseudocolored based on confocal sections to distinguish three retinal plexuses; superficial shown in green, intermediate in cyan, and deep in red. Vascular density was assessed as percentage of total area. **p < 0.01, *p < 0.05, ns: not significant. Two-tailed paired t-test was used for analysis. **(B)** Retinas were stained with antibody against Collagen IV (Red) and with Isolectin B4 (Green) to identify acellular capillaries and compared their frequency in non-diabetic to diabetic. **p < 0.01. **(C)** Retinas were stained with SA-β-Galactosidase to identify senescent cells by the blue staining. Counterstaining with Isolectin B4 in green to show senescent cells within the retinal vasculature. Scale bars: 50 µm.

**FIGURE 5:**
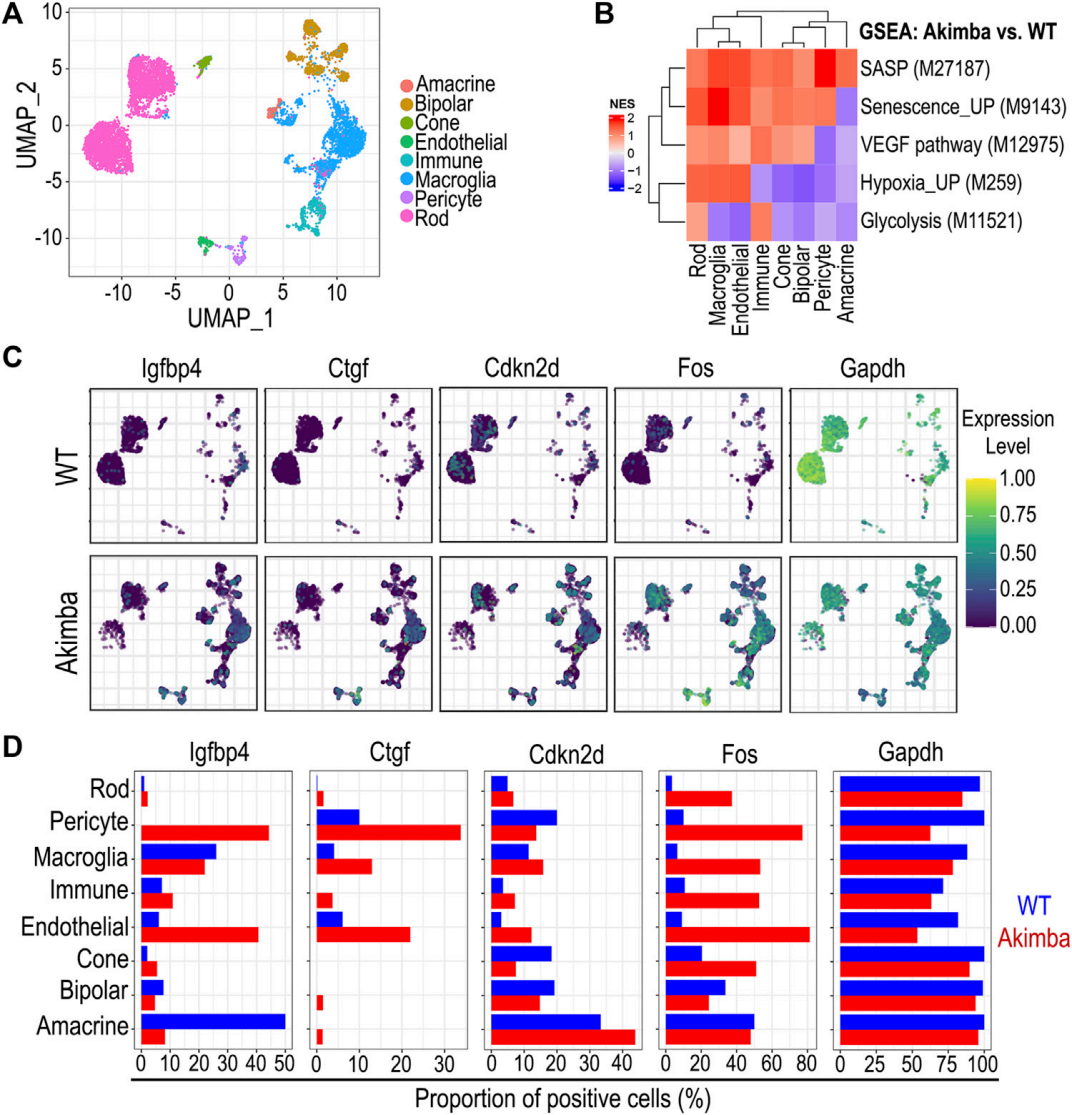
Single Cell RNA sequencing analysis of the Akimba mouse model for diabetic retinopathy. **(A)** Clustering of retinal cells to distinguish cell types. **(B)** Gene set enrichment analysis comparing diabetic Akimba retinas vs. wild type controls per cell type. **(C)** Expression level for genes from SASP, senescence, and glycolysis signatures to highlight differences at transcript level. **(D)** Bar plots quantifying proportion of positive cells for transcripts shown in UMAPs.

We performed histological analysis of 6–9-months retinas from diabetic db/db mice and compared them to age-matched control db/+ mice. Diabetic mouse db/db retinas showed a significantly diminished vascular density in the intermediate and deep retinal plexuses when compared to non-diabetic mouse retinas (Figure 4A). A hallmark of diabetic vasculopathy in the retina is the presence of increased acellular capillaries, characterized as Collagen IV+ Isolectin B4- structures within the retinal vascular tree. Retinas from db/db mice exhibited a significant increase in acellular capillaries when compared to non-diabetic retinas (Figure 4B). Tissue analysis and staining for SA-β-Gal identified positive cells in db/db retinas. Morphological evaluation and staining with Isolectin B4 identified these senescent cells as endothelial (Figure 4C) and microglia-like cells (Supplementary Figure S7). Furthermore, we quantified the percentage of SA-β-Gal positive area within the vasculature, and found that while in the non-diabetic mice, SA-β-Gal was negligible, in the 6–9 months db/db retinas, they accounted for 7% on average (Supplementary Figure S8). To extrapolate our results to another *in vivo* model of diabetic retinopathy, we used publicly available single cell RNA sequencing data from 12-weeks Akimba and age-matched control mouse retinas (Van Hove et al., 2020). The Akimba mouse model presents with retinal neovascularization on a hyperglycemic background and is generated from crossbreeding the Akita hyperglycemic mice with the Kimba VEGF overexpressing mice (Rakoczy et al., 2010). The combination of genotypes leads to more severe retinal vascular pathology, neovascularization, fibrosis, and edema, which mimics key aspects of human diabetic retinopathy. We identified 8 clusters that corresponded to specific retinal cell types (Figure 5A). The endothelial cell cluster had 5,445 transcripts that were evaluated, and we found 66 differentially expressed genes (DEGs), when comparing Akimba vs. control retinas. To understand the relative contribution of senescence and glycolysis in this model, we performed GSEA analyses comparing diabetic (Akimba) vs. age matched non-diabetic retinas. The comparisons showed enrichment of SASP (M27187) and senescent (M9143) gene signatures in endothelial cells. Interestingly, glycolysis-related genes (M11521) showed negative enrichment values in endothelial cells from diabetic retinas, when compared to non-diabetic controls (Figure 5B). Retinas from Akimba mice showed both higher expression and number of positive cells for senescence (*cdkn2d, fos*) and SASP (*igfbp4, ctgf*) associated genes. On the other hand, glycolysis (*gapdh*) genes were downregulated in diabetic animals (Figures 5C,D). In alignment with our previous data, Akimba diabetic retinas show accumulation of cellular senescence and impaired metabolic function. We also evaluated gene expression changes in senescence biomarkers p21, p16, and p53 (Supplementary Figure S9). An increase of p21 gene expression was evident in pericytes and macroglia, but not in endothelial cells, when comparing Akimba with control retinas. Interestingly >60% of retinal endothelial cells expressed p21 gene, irrespective of being a diabetic animal or not. Expression of p16 gene was only present in cells from Akimba retinas, however at low frequency (<3%) in most retinal cells, except pericytes (∼17%). Expression of p53 gene increased in all cells from Akimba retinas when compared to controls, except in bipolar cells. In endothelial cells, p53 increased by two-fold from ∼8% to ∼16%. In addition, we scored gene signatures for senescence (FRIDMAN_SENESCENCE_UP M9143) and the SASP (REACTOME_SENESCENCE_ASSOCIATED_SECRETORY_PHENOTYPE_SASP M27187) in Akimba retinas compared to WT controls (Supplementary Figure S10). Normalized enrichment scores indicated that the Fridman senescence signature had higher scores in pericytes (NES 1.99), immune cells (NES 1.90), macroglia (NES 1.49) and endothelial cells (NES 1.37), while neural cells did not show any enrichment. The SASP signature highlighted largest enrichment scores in pericytes (NES 2.14), macroglia (NES 2.02), and endothelial cells (NES 1.72). These results underscored the importance of cell type-specific senescence programs associated with distinct responses to the diabetic microenvironment. Our results assessing retinas in two distinct diabetic murine models, db/db and Akimba, demonstrated at the tissue histological and transcriptomics levels, respectively, that cellular senescence in retinal endothelial cells was accelerated with diabetes.

## 4 Discussion

Diabetic tissues exhibit accumulation of senescent cells; therefore, a link between senescence and diabetes has been proposed (Palmer et al., 2019). It has also been suggested that the diabetic microenvironment including high glucose, high lipids, and chronic low-grade inflammation may facilitate senescence generating a pathogenic loop, where senescent cells are both cause and consequence of diabetic complications (Palmer et al., 2015). Our study confirmed that high glucose was sufficient to induce premature senescence of HRMECs *in vitro*. This senescent phenotype induced by high glucose was coupled with impairment of endothelial function and decreased glycolysis. Our *in vitro* model is a well-accepted experimental approach to investigate cell senescence (Medina et al., 2013; Chan et al., 2022). We combined the classic replicative senescence model with additional chronic exposure to high glucose to mimic a diabetic microenvironment. There has been extensive debate about the physiological relevance of replicative senescence; however, cumulative evidence has demonstrated that cell replicative life span is genetically related to organism life span (Campisi, 1996). Nevertheless, some limitations of the model include the lack of other relevant cell types, constant exposure to growth cues likely lacking *in vivo*, and the requirement to frequently trypsinize cells during passages. Notwithstanding the simplicity of our experimental model, high glucose levels alone accelerated the senescence programme in HRMECs, and it is expected that in combination with other factors such as inflammation, hyperlipidaemia, oxidative stress, and advanced glycation end products (AGEs), as a complex diabetic microenvironment, consequent senescence will be triggered earlier than the 4 weeks required for high D-glucose.

Some studies have found that high glucose promotes endothelial proliferation, invasion and angiogenesis (Li et al., 2016; Fang and Chang, 2021; Wang et al., 2022; Yang et al., 2022). Our investigation focussed on HRMECs cultured under high glucose conditions for over 60 days, while most previous studies exposed cells for a maximum of 2 weeks. Our results identified significant changes in HRMECs growth, senescence, and endothelial function from 4 weeks onwards. We also found that the high glucose-induced premature senescence was associated with lower cumulative population doublings. This lower Hayflick limit in high glucose treated HRMECs suggests that the accelerated senescence was unlikely a consequence of increased proliferation at early stages.

Senescent cells show an enhanced glycolytic state (Wiley and Campisi, 2016; Sabbatinelli et al., 2019), which we confirmed in replicative senescent HRMECs (data not shown); however, when the high glucose environment was added to the *in vitro* senescence model, we found a significant decrease in glycolysis. Such findings suggest that cellular bioenergetics in senescent endothelium under diabetic conditions is different to senescence under physiological aging. These results agree with a study that evaluated glucose metabolism in diabetic rat retinas and found a decline in the glycolytic flux in the diabetic retinas when compared to controls (Ola et al., 2006). Moreover, a recent study has demonstrated decreased glycolysis and diminished PFKFB3 in 7-month diabetic Akita mice, compared to age-matched controls (Shosha et al., 2022), in alignment with our results. This is highly relevant because endothelial cells utilize glycolysis as their primary ATP producing mechanism, and our results showed that high glucose-induced senescent HRMECs exhibited significant decreased glycolysis. A negative feedback mechanism has been shown in HUVECs, where high glucose suppressed hexokinase 2 expression leading to apoptosis by impairing the interaction between Bax and VDAC1 (Zhang et al., 2019). Although we did not find any changes in hexokinase 2 expression or activity in the high glucose treated HRMECs, we observed a decrease in glucose transporters GLUT1, GLUT3, and the glycolytic enzyme PFKFB3, which suggest negative feedback in our experimental model, in response to the high availability of glucose. Similar findings indicating a decrease in GLUT1 expression using *in vivo* and *in vitro* models of diabetic retinopathy were reported to be due to increased degradation by ubiquitination of GLUT1 (Fernandes et al., 2004). All this evidence suggests that, although the precise molecular mechanisms may differ across the experimental models, endothelial cells consistently respond to the high glucose environment by limiting glucose utilization.

p16^INK4A^ positive senescent endothelial cells accumulate in human retinas with proliferative diabetic retinopathy (Crespo-Garcia et al., 2021). This was further confirmed by scRNA-seq in a mouse model of oxygen-induced retinopathy (OIR), which identified senescence signatures in many retinal cell types within the ischemic retina that include astrocytes, pericytes, endothelial cells, and Muller glia. This is in agreement with our analysis of the Akimba diabetic mouse model which also showed increase of p16 in retinal pericytes under diabetic conditions. Importantly, analysis of the Akimba scRNAseq dataset also highlighted an enrichment for senescence gene signatures in pericytes, macroglia, and endothelial cells. The transcriptome analysis at single cell resolution enabled us to find major differences in gene signatures between specific cell types within the Akimba mouse retinas. For example, results indicated that pericytes were the retinal cells with highest scores for senescence gene signatures, while macroglia (retinal Muller cells and astrocytes) and pericytes exhibited the highest scores for SASP, in contrast to neural cells like rods and bipolar cells whose gene signatures for senescence or SASP did not change in the diabetic setting. While our study focused on endothelial cells, results highlighted that all cells within the retinal tissue should be evaluated as diabetes will likely impair their cell-cell cross communication. Our histological analysis in db/db retinas also identified endothelial cells and microglia-like cells positive for senescence marker SA-β-Galactosidase. The presence of increased cellular senescence in diabetic retinas provides a rationale for the use of senolytics. In mice, senolytics that inhibit Bcl-xl were effective in reducing pathological neovascularization and enhancing vascular repair in the OIR model (Crespo-Garcia et al., 2021). These findings open therapeutic opportunities for diabetic eye disease, and importantly, a retrospective clinical study in 28 glaucoma patients indicated that senolytics are not toxic in the eye and can be neuroprotective (El-Nimri et al., 2020).

Our results highlighted that at 4 weeks of high glucose exposure, HRMECs showed a robust increase in their senescence phenotype, impaired endothelial function, and diminished glycolysis, but, on the other hand, changes at the transcriptome level and biochemicals assays were less evident. This could be explained by the biological variability, shown by the cells from different donors used for this study. In addition, it is possible that molecular changes at the gene expression level precede the senescent and functional phenotype, and transcriptome changes may be clearer at an earlier stage, during establishment of the senescent and dysfunctional phenotype (Ye and Steinle, 2015; Zhao et al., 2016).

In summary, we show evidence for high glucose as an accelerator of the senescence program in HRMECs, with impaired endothelial function and glycolysis. We also described the presence of a senescence gene signature and senescent cells in the retinas of two mouse models of diabetic retinopathy, the db/db and the Akimba. These results support the development of novel strategies to treat diabetic retinopathy by targeting senescent endothelial cells, by using senolytics or modulating disease-induced shifts in endothelial glycolysis.

## Data Availability

The datasets presented in this study can be found in online repositories. The names of the repository/repositories and accession number(s) can be found below: https://www.ncbi.nlm.nih.gov/geo/, GSE199548, and https://www.ebi.ac.uk/arrayexpress/search.html?query=akimba, E-MTAB-9061.
