# Supplementary material for: Long term high glucose exposure induces premature senescence in retinal endothelial cells
Source: Front Physiol. 2022 Aug 26;13:929118. doi: 10.3389/fphys.2022.929118 (PMC9459081; doi:10.3389/fphys.2022.929118)
Supplement: Supplementary file 1 [file DataSheet1.pdf]

Supplementary Material

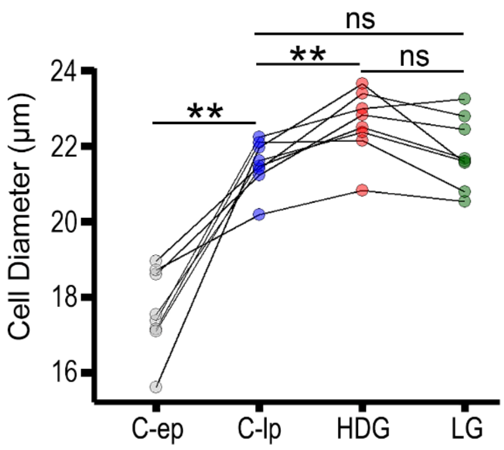

Supplementary Figure 1. Cell diameter measured using the CASY system. **p<0.01, ns: not significant. One-way ANOVA, with Tukey's post-hoc analysis was used. C-ep, control early passage (5 mM D-glucose); C-lp, control late passage (5 mM D-glucose); HDG, high D-glucose (25 mM D-glucose); LG, osmotic control (5 mM D-Glucose + 20 mM L-glucose).

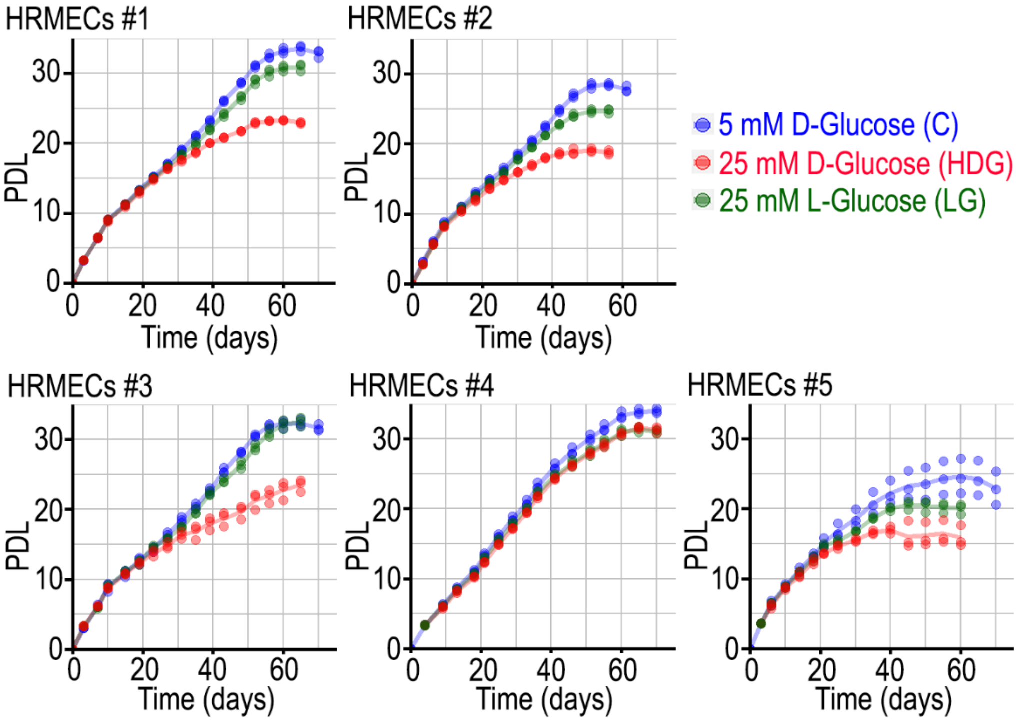

Supplementary Figure 2. Growth curves for human retinal microvascular endothelial cells (HRMECs) exposed to a high glucose environment of 25mM D-glucose (HDG), compared to controls 5mM D-glucose, and osmotic control L-Glucose. Data for HRMECs from five different donors. Experimental groups within each biological replicate had three technical replicates.

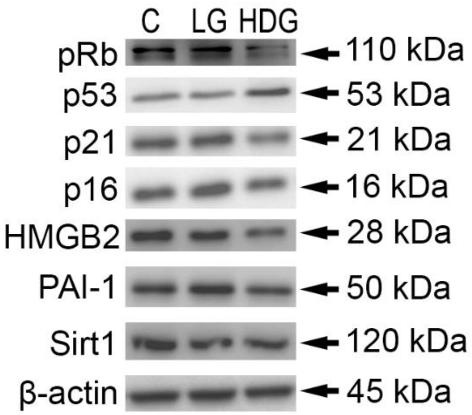

Supplementary Figure 3. Western blotting to assess expression of senescence related proteins in HRMECs.

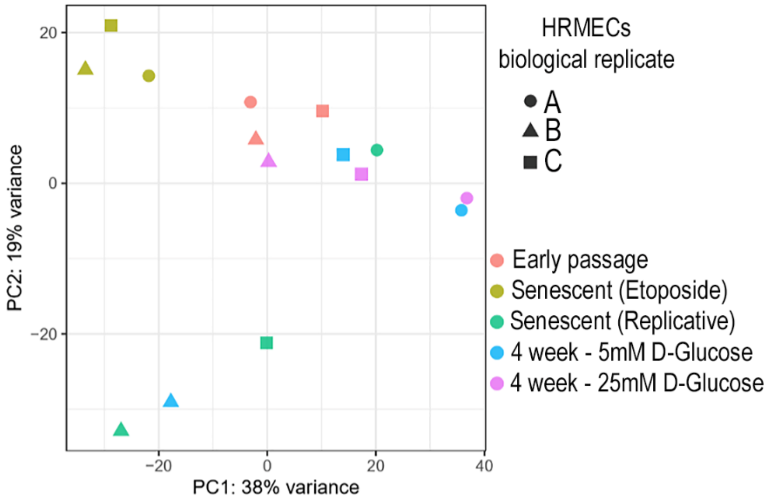

Supplementary Figure 4. Principal component analysis (PCA) of RNA sequencing for our 4-week HRMEC model exposure to 25mM D-glucose. Controls include non-senescent early passage cells, and senescent HRMECs from two senescence models (Replicative and Stress-induced with Etoposide).

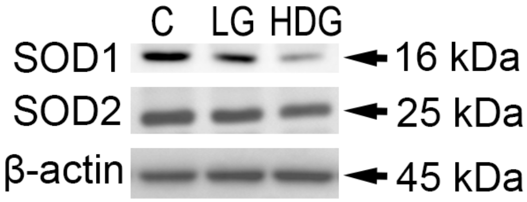

Supplementary Figure 5. Western blotting to assess expression of superoxide dismutase proteins (SOD) in HRMECs.

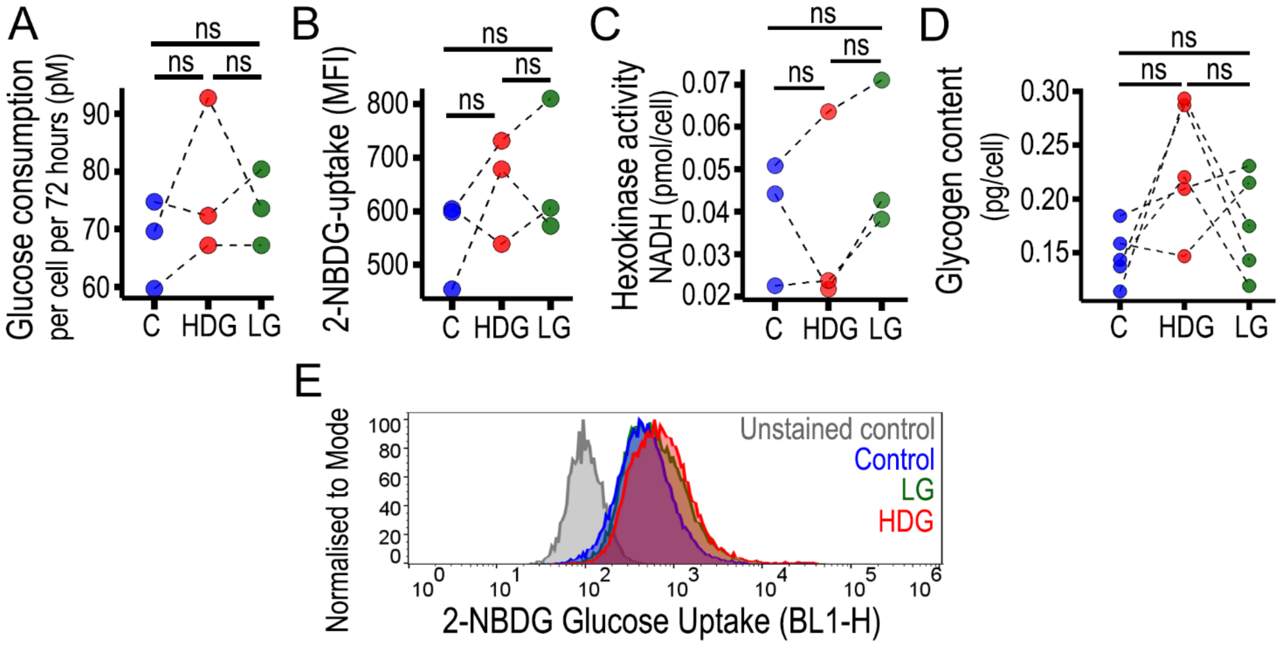

Supplementary Figure 6. (A) Glucose consumption measurements. **(B)** Glucose uptake assay using 2-NBDG and flow cytometry. MFI: Median Fluorescence Intensity. **(C)** Hexokinase activity evaluation. **(D)** Glycogen content measurement. **(E)** Histograms depicting raw data for 2-NBDG assay. ns: not significant.

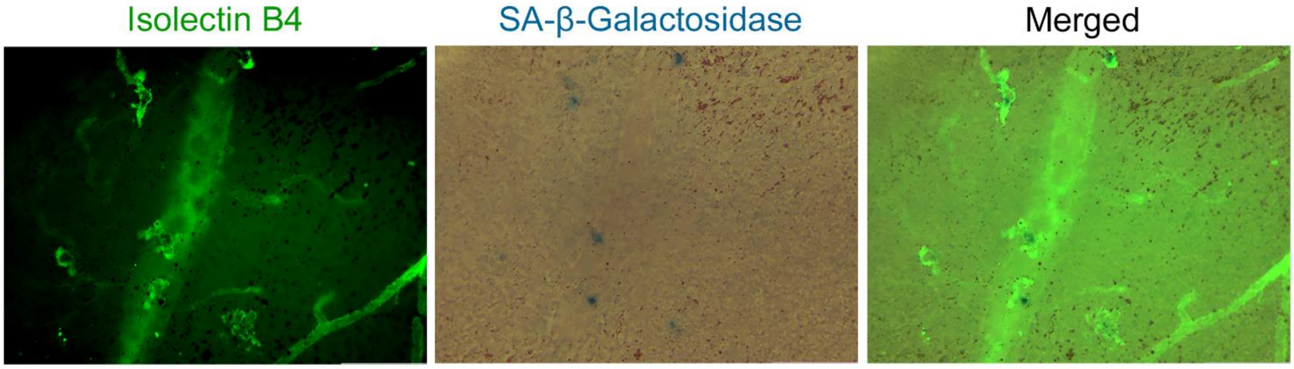

Supplementary Figure 7. Retinal microglia-like cells showed positivity for senescence biomarker. Representative image of db/db retina stained with Isolectin B4 in green and SA-β-Galactosidase in blue. Isolectin B4 stains myeloid cells in addition to endothelial cells, and in the retina, these cells represent microglia.

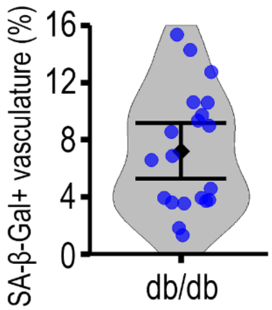

Supplementary Figure 8. Quantification of SA-β-Gal positive vasculature in retinal flat-mounts from 6- and 9-month db/db mice. SA-β-Gal signal was quantified as percentage in relation to the total retinal vasculature (length). Each data point represents a retinal image. Six retinal flat mounts were used.

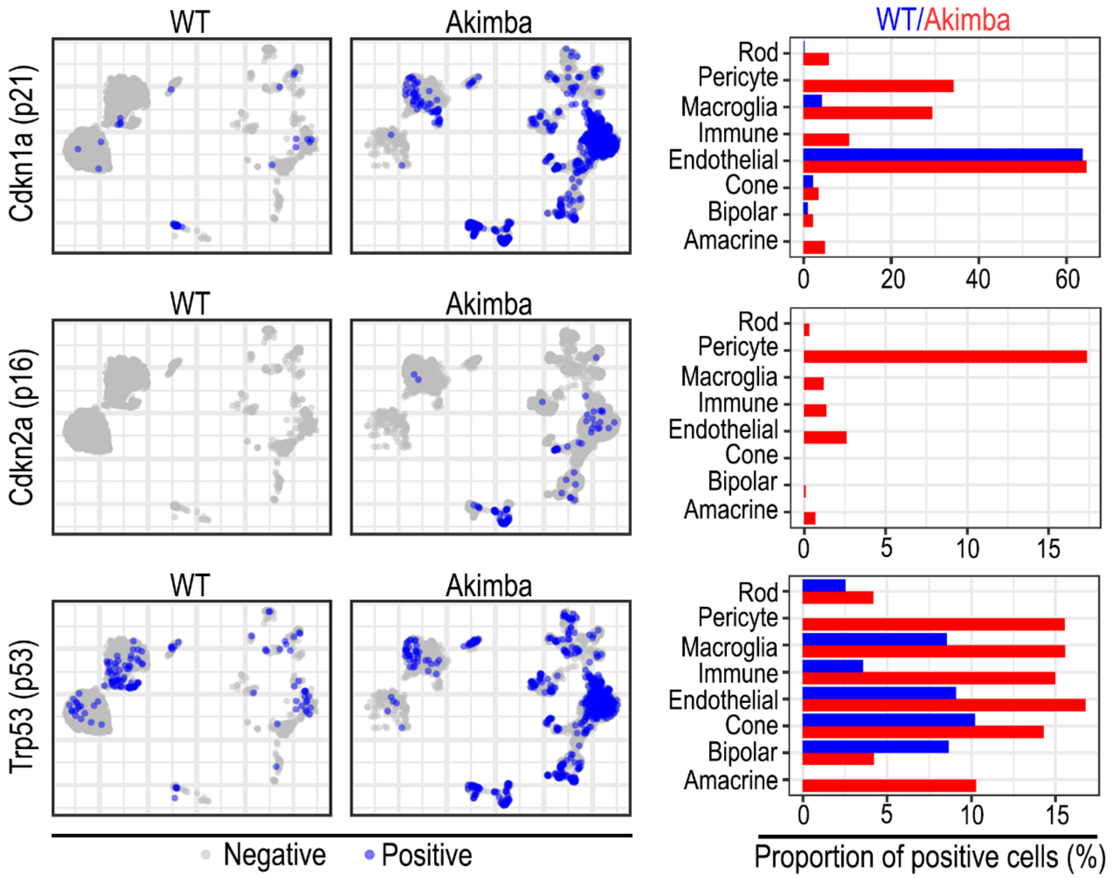

Supplementary Figure 9. Single-cell RNA sequencing analysis of the Akimba mouse model for senescence biomarkers p21, p16 and p53. Expression level for genes of interest shown as positive in blue or negative in grey. Bar plots quantifying proportion of positive cells for transcripts shown in UMAPs.

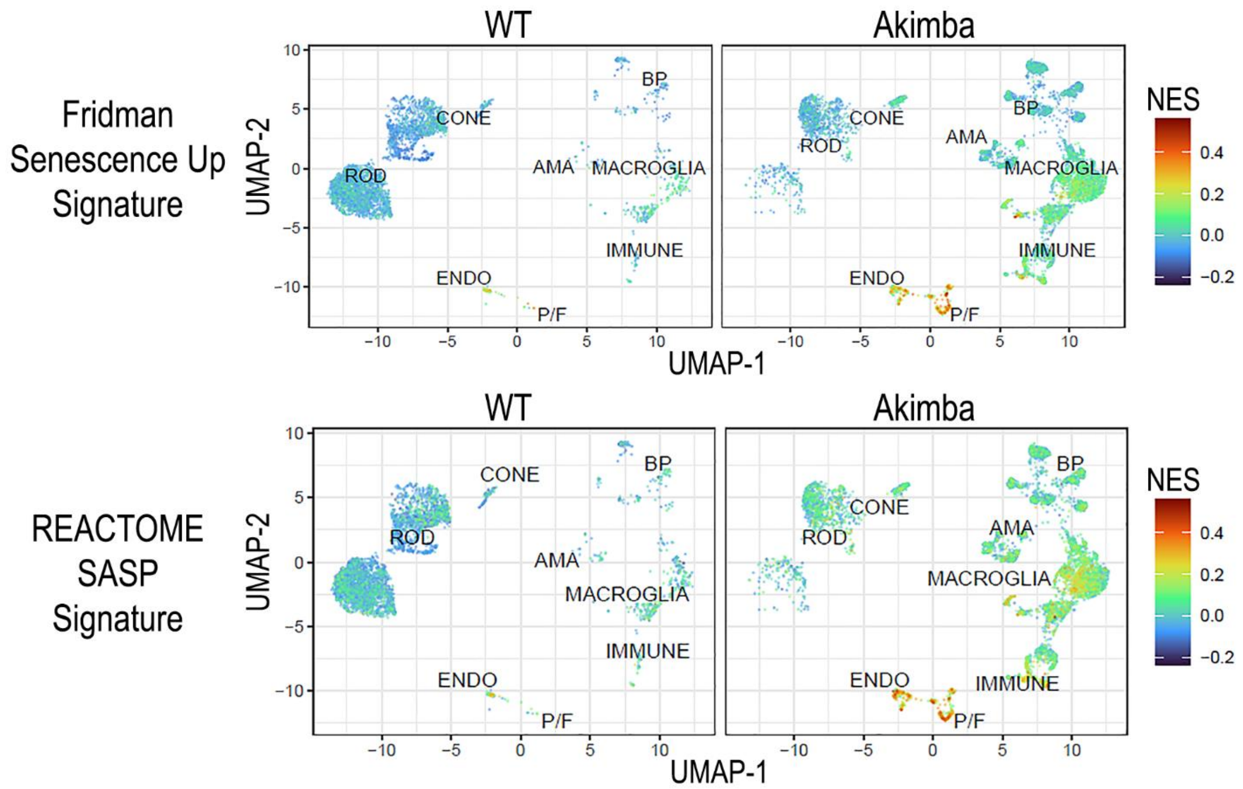

Supplementary Figure 10. Single-cell RNA sequencing analysis of the Akimba mouse model compared to WT control mouse retinas for gene signatures related to senescence and SASP. Normalized enrichment scores (NES) are depicted in turbo color scale bar and applied to UMAPs.
